# Health Literacy Levels of Patients With Juvenile Idiopathic Arthritis and Their Parents

**DOI:** 10.1111/hex.14117

**Published:** 2024-06-16

**Authors:** Yahya Doğan, Nur Banu Karaca, Sinan Buran, Ege Nur Atabey Gerlegiz, Emil Aliyev, Yağmur Bayındır, Yelda Bilginer, Edibe Ünal, Seza Özen

**Affiliations:** ^1^ Department of Physical Medicine and Rehabilitation University of Health Sciences Kocaeli Derince Training and Research Hospital Kocaeli Turkey; ^2^ Department of Physiotherapy and Rehabilitation, Faculty of Physical Therapy and Rehabilitation Hacettepe University Ankara Turkey; ^3^ Division of Pediatric Rheumatology, Department of Pediatrics, Faculty of Medicine Hacettepe University Ankara Turkey

**Keywords:** education, health related quality of life, juvenile idiopathic arthritis, parents, rheumatic diseases

## Abstract

**Background:**

The aim of this study was to reveal the relationship between the health literacy (HL) levels of children with juvenile idiopathic arthritis (JIA) and their parents, and the general health status and physical performance of the children.

**Methods:**

This study included 79 children aged 9–18 years with a diagnosis of JIA and one of their parents. HL levels were evaluated with the Turkish version of the Health Literacy for School‐Aged Children and Turkish Health Literacy‐32 (THL‐32) for children and Adult Health Literacy Scale (AHLS) for their parents. The Childhood Health Assessment Questionnaire (CHAQ), 6‐minute walk test (6‐MWT), 10‐meter walking test (10‐MWT) and 10‐stair climbing test (10‐SCT) was used to evaluate the children. Juvenile Arthritis Biopsychosocial Questionnaire (JAB‐Q) was used to assess the children's and parents' psychosocial status and perception of health.

**Results:**

HL levels of patients with JIA were 16.5% low HL, %55.7 moderate HL and 27.8% high HL. According to THL‐32 scale score, HL level of parents were as follows: inadequate, 3.8%; problematic, 22.8%; sufficient, 34.2%; and excellent, 39.2%. Children's HL levels increase positively as they get older, and no significant relationship was found with other parameters. The AHLS, CHAQ and JAB‐Q scores were better in the group with higher education levels of the parents. No statistically significant association was found between the HL of the children and that of the parents.

**Conclusion:**

In our study, it was found that the high education levels of the parents positively affected the quality of life and physical condition of their children and parental HL levels. In addition, it was shown that the HL levels of children with JIA were not statistically related to other parameters.

**Patient or Public Contribution:**

Children diagnosed with JIA and one of their parents actively participated in the study. Feedback from children and families provided important information about obtaining and using HL information before and during the study. The importance of therapy programs and information focusing on the patient and their family, as well as the inter‐multidisciplinary approach, in combating a chronic disease at an early age was reinforced by the feedback received from patients and their families.

## 1 Introduction

Juvenile idiopathic arthritis (JIA) is a chronic rheumatological disease characterized by persistent joint inflammation [1]. The term juvenile is used because it starts before the age of 16, and the term idiopathic is used because the exact cause is unknown [2]. This disease causes paediatric patients to encounter health problems at an early age and therefore struggle with more questions and problems than their peers [3]. At this point, it is important that both children and their families have access to adequate and accurate information about health and that they can use this information [4]. Access to health‐related information has brought the concept of ‘health literacy’, which has become more important in recent years, to the agenda [5]. According to the World Health Organization (WHO), health literacy (HL) is the cognitive‐social skills and motivation levels of individuals to access, understand and use information to protect and improve their health [6]. Although there are various questionnaires to assess HL, very few of them have been translated into Turkish and validity studies have been conducted [7, 8]. There is much less worldwide research on children's HL.

The main aim of this study is to reveal the HL levels of children with JIA and their caregivers by using the Turkish Health Literacy‐32 (THL‐32), Adult Health Literacy Scale (AHLS) and Turkish version of the Health Literacy for School‐Aged Children (HLSAC‐T), the validity and reliability studies of which have been done before [9, 10, 11]. In addition, revealing the relationship between HL and the health status, quality of life and physical–functional status of children with JIA is the secondary main aim of the study. Thus, it is aimed to display a holistic approach by evaluating the relationship between HL and general health status and physical capacity.

## 2 Materials and Methods

### 2.1 Study Population

This cross‐sectional study was conducted between September and December 2022 in a tertiary care university hospital, in collaboration with the Paediatric Rheumatology outpatient clinic and the Rheumatological Rehabilitation unit. Inclusion criteria were a confirmed JIA diagnosis according to the International League Against Rheumatism (ILAR) criteria, aged between 9 and 18 years. Children and one of their parents were invited to study. Subjects were excluded if they had comorbidities associated with limitations in mental or ambulation ability, had malignancies and were unable to understand and complete the questionnaires.

### 2.2 Procedure

Children and their parents were recruited consecutively during routine clinic visits. Data were collected from children and accompanying parents. Demographic data, clinical findings, laboratory findings and previous treatment information were recorded retrospectively from patient files and by asking the participants themselves. The study was approved by the Ethical Committee of Hacettepe University (Approval: GO‐22/397). Written informed consent was obtained from all participants before study enrolment.

### 2.3 Measures

Sociodemographic characteristics of the child and parents (e.g., age, gender, body mass index, disease characteristics, medical treatment, educational status) were recorded at baseline. The Juvenile Arthritis Disease Activity Score‐71 (JADAS‐71) (for oligoarticular and polyarticular subtypes) and the Bath Ankylosing Spondylitis Disease Activity Index (BASDAI) (for enthesitis‐related arthritis subtype) were used to evaluate the disease activity of patients with nonsystemic JIA. Parents' educational status was classified as low (primary and secondary school) and high (high school and above) by their last graduation. Based on the aims and hypotheses of this study, evaluations were made with the following questionnaires and clinical tests.

#### 2.3.1 Childhood Health Assessment Questionnaire (CHAQ)

Functional ability was assessed with the Turkish version of the CHAQ. This questionnaire evaluates the child's functional level in eight areas of daily life (dressing/grooming, arising, eating, walking, hygiene, reach, grip and activities), for the past week [12]. The score ranges from 0 to 3, with high values indicating poor physical function. The CHAQ score of ≥1 means severe disability. In addition, regardless of this score, there are two visual analogue scales (range: 0–10) at the end of the questionnaire to evaluate the level of pain (CHAQ—pain evaluation) and the overall effect of the disease on the child's life (CHAQ—general evaluation).

#### 2.3.2 Juvenile Arthritis Biopsychosocial Questionnaire (JAB‐Q)

This questionnaire evaluates the problems experienced by both the child and the parent from a biopsychosocial perspective [13]. It includes two separate forms for the child (patient) and parent (family) to complete. The child form consists of questions evaluating parameters such as pain intensity, disease activity, joint status, functional assessment, psychosocial status assessment, school status and fatigue, and is scored between 0 and 52 points. The family form includes parameters such as the parent's perception of health and psychosocial status and scores between 0 and 38. Forms can give both total scores and separate scores for subparameters. Higher scores indicate worse results.

#### 2.3.3 Children's Depression Inventory (CDI)

The Turkish version of the CDI, consisting of 27 multiple‐choice items questioning depressive symptoms, was used to evaluate the depression level of children [14]. Scores range from 0 to 54, and a high score indicates a bad depressive state. A score of ≥19 is considered clinically significant.

#### 2.3.4 HLSAC‐T

The HLSAC‐T scale was used to measure the subjective HL of children [11]. The scale consists of 10 items containing different dimensions of HL (theoretical knowledge, practical knowledge, critical thinking, self‐awareness and citizenship) and scores between 10 and 40. A high score indicates that the child's HL level is good; HL level is considered in four categories according to the score obtained: 10–25 points indicate low HL, 26–35 points indicate moderate HL and 36–40 points indicate high HL.

#### 2.3.5 6‐Minute Walk Test (6‐MWT)

The 6‐MWT, which is frequently preferred in various paediatric and adult diseases and measures the distance people can walk in 6 min, was used to measure submaximal exercise capacity. The test was performed using standard instructions defined by the American Thoracic Society [15].

#### 2.3.6 10‐Meter Walk Test (10‐MWT) and 10‐Stair Climbing Test (10‐SCT)

The participants' 10‐MWT and 10‐SCT times were recorded in seconds. The tests were performed thrice, and the mean value was recorded [16].

#### 2.3.7 Turkish Health Literacy‐32 (THL‐32)

It is a 32‐item HL scale developed based on the European Health Literacy Survey (HLS‐EU) Consortium 2012 [9]. The scale is scored between 0 and 50 points. A high score indicates good HL. HL level is considered in four categories according to the score obtained: 0–25 points indicate inadequate HL, >25–33 points indicate problematic HL, >33–42 points indicate sufficient HL and >42–50 points indicate excellent HL.

#### 2.3.8 Adult Health Literacy Scale (AHLS)

The AHLS developed in the Turkish population was used to determine the HL of the patients' parents. This scale consists of 23 questions questioning both subjective and objective HL [10]. The scale includes yes/no questions, fill‐in‐the‐blank, multiple‐choice and matching questions. The score on the scale varies between 0 and 23. High scores indicate good HL level.

### 2.4 Statistical Analysis

All statistical analyses were performed using the IBM SPSS Statistics version 23.0 software package. Histogram graphics and the Kolmogorov–Smirnov test were used to test for normality. Descriptive statistics were presented as median/interquartile range (IQR: 25th–75th percentile) or mean/standard deviation for quantitative variables according to data distribution, and number/percentage (%) for qualitative variables. A *p* value less than 0.05 was considered to be statistically significant. Kruskal–Wallis and Mann–Whitney *U*‐tests were used to compare medians in subgroup analyses. In the post hoc analysis, Bonferroni adjustment for multiple comparisons was used. The correlations were assessed using Spearman's correlation analysis. The correlation coefficient was classified as weak (*r* = 0.10–0.39), moderate (*r* = 0.40–0.69), strong (*r* = 0.70–0.89) and very strong (0.90–1.00).

## 3 Results

### 3.1 Patients' Characteristics

Seventy‐nine patients with JIA and one of their parents were included in the study. The patients' mean age was 13.67 ± 2.80 years, and 60.8% of them were female. The mean disease duration was 6.75 ± 4.28. The JIA subtypes were as follows: 39 (49.4%) had oligoarticular JIA; 15 (19.0%) had polyarticular JIA; 19 (24.1%) had enthesitis‐related arthritis; and 6 (7%) had systemic JIA. Sixteen (20.3%) patients had regular exercise/sports habits at least 2 days a week. The parents' median age was 41.84 (IQR: 38–46) years. The family questionnaires were completed by mothers (74.7%) and fathers (25.3%). The detailed characteristics of the participants are shown in Table 1.

**Table 1:** Characteristics of the participants with juvenile idiopathic arthritis and their parents.

Patient characteristics	*n* = 79	
Age (year): mean ± SD	13.67 ± 2.80	14 (11–16)
BMI (kg/m^2^): mean ± SD	19.53 ± 3.34	19.15 (17.51–22.06)
Gender (*n*, %)	
Female	48 (60.8%)	
Male	31 (39.2%)	
Regular exercise/sports habit	
Yes	16 (20.3%)	
No	63 (79.7%)	
JIA characteristics	
Duration of disease (year): mean ± SD	6.75 ± 4.28	7 (3–10)
CRP (mg/dL)	0.257 (0.175–1.0.571)	
ESR (mm/h)	8 (3–15)	
JADAS‐71^[Link]^, ^a^	0 (0–6.70)	
BASDAI^b^	2.01 (0–2.77)	
JIA subtype (*n*, %)	
Oligoarticular JIA	39 (49.4%)	
Polyarticular JIA	15 (19.0%)	
Enthesitis‐related arthritis	19 (24.1%)	
Systemic JIA	6 (7%)	
Treatment, at time of visit (*n*, %)	
NSAID use	10.3%	
DMARD use	22.4%	
Biologic agents use	48.3%	
Drug‐free	25.9%	
Family characteristics	
Age (year)	41.84 (38–46)	
BMI (kg/m^2^)	26.70 (24.09–28.36)	
Parents	
Mother	59 (74.7%)	
Father	20 (25.3%)	
Parental education status	
Low	
Primary school	24 (30.4%)	
Secondary school	11 (13.9%)	
High	
High school	22 (27.8%)	
Associate degree	4 (5.1%)	
Bachelor's degree	16 (20.2%)	
Master's degree	1 (1.3%)	
Doctor of Philosophy	1 (1.3%)	

*Note:* Values are medians (25th, 75th percentiles [IQR: interquartile range]), unless otherwise stated.

Abbreviations: BASDAI = Bath Ankylosing Spondylitis Disease Activity Index, BMI = body mass index, CRP = C‐reactive protein, DMARD = disease‐modifying antirheumatic drugs, ESR = erythrocyte sedimentation rate, JADAS‐71 = Juvenile Arthritis Disease Activity Score in 71 joints, JIA = juvenile idiopathic arthritis, NSAID = nonsteroidal anti‐inflammatory drugs.

^a^ *n* = 61.

^b^ *n* = 18.

### 3.2 HL and Related Factors

Thirty‐five parents (44.3%) had low, and 44 parents (55.7%) had high education levels. Following the THL‐32 scale scoring, level of parents' HL resulted in the following percentages: inadequate, 3.8%; problematic, 22.8%; sufficient, 34.2%; and excellent, 39.2%. From a total of 79 patients with JIA, 16.5% had low HL, %55.7 had moderate HL and 27.8% had high HL. Details regarding the educational status of the parents and the HL levels of all participants are shown in Table 2. None of the analysed data showed statistical differences in terms of HL levels (*p* ^0,1^ > 0.05) except for patients' age (*p* ^0^ < 0.05) (Table 3).

**Table 2:** Educational status of the parents and the health literacy levels of all participants.

	Education status		
Parents (*n* = 79)	Low education level	High education level	Total (*n*, %)
THL‐32 subgroups (*n*, %)			
Inadequate HL	3	0	3 (3.8)
Problematic HL	8	10	18 (22.8)
Sufficient HL	13	14	27 (34.2)
Excellent HL	11	20	31 (39.2)
Total N	35 (44.3)	44 (55.7)	79 (100)
AHLS, median (IQR)	13 (10–15)	16 (14.8–18)	15 (13–17)

Abbreviations: AHLS = Adult Health Literacy Scale, THL‐32 = Turkish Health Literacy‐32.

**Table 3:** Comparison of characteristics and outcome measures by health literacy level in children with JIA and their parents.

		HLSAC‐T subgroups				THL‐32 subgroups				
Patient characteristics, mean ± SD		Low	Moderate	High	*p* ^0^	Inadequate	Problematic	Sufficient	Excellent	*p* ^1^
Age (year)		13.08 ± 2.98	13.05 ± 2.44	15.27 ± 2.88	0.009	48.33 ± 5.86	41.7 ± 6.65	41.9 ± 7.18	41.25 ± 5.38	0.268
Duration of disease (year)		5.92 ± 3.84	6.84 ± 4.76	7.05 ± 3.55	0.782	8.33 ± 5.13	6.89 ± 4.97	5.44 ± 4.55	7.65 ± 3.36	0.184
Measure		*p* ^0^				*p* ^1^				
CHAQ			
Pain (0–10)	1.1 (0–3.8)	0.856				0.474				
General (0–10)	2.2 (0.8–4.8)	0.841				0.568				
Total (0–3)	0.125 (0–0.5)	0.987				0.682				
JAB‐Q Child Form			
Pain severity (0–10)	2.03 (0–3)	0.544				0.784				
Disease activity (0–10)	2 (0–3)	0.427				0.937				
Functional status (0–66)	4.31 (1–6)	0.650				0.422				
Fatigue total (0–16)	11 (5–14)	0.519				0.120				
Child—Psychosocial (0–52)	12.54 (8–15)	0.166				0.801				
Child form—Total (0–164)	25.8 (14–31)	0.321				0.734				
CDI (0–54)	10 (6–14)	0.112				0.585				
HLSAC‐T (10–40)	32 (28–36)	—				0.488				
Clinical tests			
6‐MWT (m)	564.66 (533–593)	0.576				0.577				
10‐MWT (s)	6 (5.44–6.53)	0.809				0.181				
10‐SCT (s)	4.23 (3.83–4.57)	0.412				0.819				
Parents	(*n* = 79)	
THL‐32 (0–50), mean ± SD	39.08 ± 7.93	0.738				—				
AHLS (0–23)	15 (13–17)	0.239				0.052				
JAB‐Q Family—Psychosocial (0–20)	5 (3–5)	0.586				0.127				
JAB‐Q Family form—Total (0–38)	10.28 (6–12)	0.378				0.363				

*Note:* Values are medians (25th, 75th percentiles [IQR: interquartile range]). unless otherwise stated.

Abbreviations: 6‐MWT = 6‐minute walk test, 10‐MWT = 10‐meter walking test, 10‐SCT = 10‐stair climbing test, AHLS = Adult Health Literacy Scale, CDI = Children's Depression Inventory, CHAQ = Childhood Health Assessment Questionnaire, HLSAC‐T = Turkish version of the Health Literacy for School‐Aged Children, JAB‐Q = Juvenile Arthritis Biopsychosocial Questionnaire, *p* ^0^ = *p* values of the Kruskal–Wallis test by comparing the HLSAC‐T subgroups, *p* ^1^ = *p* values of the Kruskal–Wallis test by comparing the THL‐32 subgroups, THL‐32 = Turkish Health Literacy‐32.

The mean ages (mean ± SD) of the children with JIA were 13.08 ± 2.98, 13.05 ± 2.44 and 15.27 ± 2.88, respectively, according to whether they were in the low, medium or high HL subgroups. The mean age of children with high HL was statistically older than the group with medium HL (*p* < 0.017 by Mann–Whitney *U*‐test).

There was no statistically significant relationship between the children's HL and parent HL (*r* = 0.094, *p* > 0.05; *r* = 0.030, p > 0.05 for AHLS and THL‐32, respectively). In addition, a weak positive correlation was determined between two different scales assessing parental HL (*r* = 0.221, *p* < 0.05) (Table 4).

**Table 4:** Correlations for health literacy measures.

*n *= 79	AHLS	HLSAC‐T
AHLS		
*r*	1.000	0.094
*p*	—	0.409
THL‐32		
*r*	0.221	0.030
*p*	0.048	0.791

Abbreviations: AHLS = Adult Health Literacy Scale, HLSAC‐T = Turkish version of the Health Literacy for School‐Aged Children, THL‐32 = Turkish Health Literacy‐32.

While there was no difference in the THL‐32 (*p* = 0.134) and HLSAC‐T (*p* = 0.996) scores depending on whether the parent had low or high education, the AHLS score was statistically significantly better in the high education group (*p* < 0.001). The CHAQ‐general score (*p* = 0.014), JAB‐Q Family form—Psychosocial (*p* = 0.015) and total scores (*p* = 0.030), and 10‐SCT time (*p* = 0.019) of the parents with high education level and their child were statistically significantly better than the low education level group (Table 5).

**Table 5:** Comparison of outcome measures by parent's education level.

	Low education level (*n* = 35)	High education level (*n* = 44)	*p* a
THL‐32	38.02 (31.54–42.77)	41.14 (33.33–47.32)	0.134
AHLS	13 (10–15)	16 (15–18)	<0.001^a^
HLSAC‐T	31 (27–36)	32 (28–35.5)	0.996
CHAQ			
Pain	1.30 (0.1–3.2)	0.9 (0–4.05)	0.209
General	3.5 (1.5–5)	1.4 (0.25–3.9)	0.014^a^
Total	0.250 (0–0.5)	0.0625 (0–0.375)	0.245
JAB‐Q			
Child—Psychosocial	12.54 (9–13)	11.50 (8–15.50)	0.424
Child form—Total	25.8 (17–28.5)	25.4 (12.5–31.5)	0.878
JAB‐Q Family—Psychosocial	5 (4–8)	4 (2.5‐5)	0.015^a^
JAB‐Q Family form—Total	10.28 (8–15)	9.5 (6–10.28)	0.030^a^
CDI	11 (8–14)	9 (5.5–13.5)	0.387
Clinical tests			
6‐MWT (m)	575 (538–604)	563.08 (526–592)	0.668
10‐MWT (s)	6.22 (5.58–6.52)	5.88 (5.43–6.61)	0.354
10‐SCT (s)	4.37 (4.03–4.67)	4.03 (3.75–4.37)	0.019^a^

*Note:* Values are medians (25th, 75th percentiles [IQR: interquartile range]).

Abbreviations: 6‐MWT = 6‐minute walk test, 10‐MWT = 10‐meter walking test, 10‐SCT = 10‐stair climbing test, AHLS = Adult Health Literacy Scale, CDI = Children's Depression Inventory, CHAQ = Childhood Health Assessment Questionnaire, HLSAC‐T = Turkish version of the Health Literacy for School‐Aged Children, JAB‐Q = Juvenile Arthritis Biopsychosocial Questionnaire, THL‐32 = Turkish Health Literacy‐32.

^a^ Mann–Whitney *U*‐test.

There was a statistically significant difference in HL scores of children with JIA according to disease subtypes (*p* < 0.05 by Kruskal–Wallis). Patients with polyarticular JIA had better HL scores compared to oligoarticular JIA (median (IQR); 36 (34–38) versus 31 (26–33); *p* < 0.008 by Mann–Whitney *U*‐test) (Table 6).

**Table 6:** Comparison of health literacy scores of children with JIA within subtypes.

JIA (*n* = 79)	O‐JIA (*n* = 39)	P‐JIA (*n* = 15)	ERA (*n* = 19)	S‐JIA (*n* = 6)	*p* ^0^	*p* ^1^	*p* ^2^	*p* ^3^	*p* ^4^	*p* ^5^	*p* ^6^
Age	12 (9–18)	14 (9–18)	16 (9–18)	12.5 (11–16)	0.002	0.025	0.001	0.468	0.149	0.157	0.031
Disease duration	7 (0–17)	6 (0–14)	4 (0–14)	10 (6–13)	0.084	0.327	0.064	0.247	0.508	0.085	0.023
Age of onset	4 (1–12)	9 (2–16)	10 (2–18)	2 (1–10)	<0.001	0.043	<0.001	0.195	0.265	0.033	0.004
HLSAC‐T	31 (26–33)	36 (34–38)	33 (28–36)	30 (27–33)	0.012	0.002	0.173	0.987	0.096	0.029	0.096

*Note:* Values are medians (25th, 75th percentiles [IQR: interquartile range]). *p* <  0.008 was defined as indicating a statistically significant difference.

Abbreviations: HLSAC‐T = Turkish version of the Health Literacy for School‐Aged Children, *p* ^0^ = *p* values of Kruskal–Wallis test by comparing the four groups (*p* < 0.05), *p* ^1^ = *p* values of Mann–Whitney *U*‐test by comparing subtype O‐JIA and P‐JIA, *p* ^2^ = *p* values of Mann–Whitney *U*‐test by comparing subtype O‐JIA and ERA, *p* ^3^ = *p* values of Mann–Whitney *U*‐test by comparing subtype O‐JIA and S‐JIA, *p* ^4^ = *p* values of Mann–Whitney *U*‐test by comparing subtype P‐JIA and ERA, *p* ^5^ = *p* values of Mann–Whitney *U*‐test by comparing subtype P‐JIA and S‐JIA, *p* ^6^ = *p* values of Mann–Whitney *U*‐test by comparing subtype ERA and S‐JIA.

## 4 Discussion

In this study, we aimed to address the HL status of the paediatric patient group with JIA, which is often overlooked. While doing this, we also examined the HL levels of their parents. More than half of the children (55.7%) diagnosed with JIA had intermediate HL levels. There is no other study investigating HL levels in patients with JIA with which this data can be compared. In a study conducted on 204 students using the same scale in Turkey [17], HL was found to be moderate at a rate of 53.4%. Although this rate is similar to our study, those with a high level of HL (41.2%) are considerably higher than our study (27.8%). This difference may be due to the fact that the aforementioned study was conducted in the general population and with more participants. Parental HL levels in this study are also similar to our study [17]. In a study conducted with 2287 children aged 13 and 15 in Italy using the same scale [18], HL levels were found to be moderate at a rate of 75%. This study included older children from the general population and had a larger number of participants. Unfortunately, there are very few studies on childhood rheumatological diseases and HL. The only study investigating HL in a group of patients diagnosed with JIA was conducted in a group of young adults aged 17–21 years, and a high rate of 98% was found [4].

About half of the patients were found to be of the oligoarticular type, similar to other studies [19, 20]. The mean disease duration was found to be 6.75 years, and we think that our patient group is sufficient to represent JIA disease and its effects.

The HL levels of children with JIA were found to be related only to age, among the factors investigated. It is expected that children will become more conscious about their health as they grow up. The rate of 98% in the study conducted with young adults is consistent with our opinion [4]. The results of the JIA‐specific health assessment questionnaire CHAQ, which assesses functionality, and the JAB‐Q, which assesses the child and family in biopsychosocial terms, were not associated with HL. In addition, the 6‐MWT, 10‐MWT and 10‐SCT were not found to be associated with HL. In a study evaluating physical activity and HL, adolescents who were members of sports clubs had a higher HL than nonmembers, regardless of age or gender [21]. In our study, those who had regular sports‐exercise habits were 20.3%. Children with rheumatological diseases may act more shyly even if they know the importance of sports and exercise for a healthy life. The low rate of doing sports may have caused the effect of functional tests on HL to not be fully observed. There are no studies investigating HL and activities of daily living in children. In a study of adults with heart disease, it was seen that patients with low HL levels had lower activities of daily living [22].

Low HL levels of parents have negative consequences for children's health [23]. Regardless of whether the child has a chronic illness or not, the child's emergency department visits are 50% more likely if the parent's HL is low [24]. It is not clear exactly what kind of relationship exists between the parent's HL level and the child's HL level [25]. In our study, we used two scales to determine the HL of parents. Although the correlations between the scales are low, both do not reveal a significant relationship with the HL status of children. In a study using the same scales as ours, a weak positive relationship was found between the HL of parents and children [17]. Before our study, we expected that as the HL of the parents increased, the HL of the children would also increase. On the contrary, parents who are more conscious about health may prevent children from taking responsibility in this regard or children may leave this task to their parents. How the health status of children is affected in such a possible scenario is a complex issue that needs further investigation. Although there is no positive or negative result between parents and children's HL in our study, we would like to underline that all possibilities are possible.

When the parameters affecting the HL of the parents are examined, their education level stands out. THL‐32 is not associated with parents' educational level, while AHLS gives better results in the higher education group. This can be explained by the fact that the questions in the AHLS scale are more objective and based on health information inquiry. The THL‐32 scale, on the other hand, may provide information about how a person sees his/her own HL level. It was also emphasized in the validity study that the THL‐32 scale made a subjective evaluation [9]. At this point, the fact that the HL scale we use for children (HLSAC‐T) includes subjective questions may have led to the absence of a relationship with AHLS. Therefore, while we recommend using the AHLS instead of THL‐32 for the assessment of adult HL, we think that HL scales should be developed for children, including objective questions compatible with their age.

A statistically significant relationship was found between the education levels of the parents and the results of the CHAQ—general evaluation and JAB‐Q family evaluation. Low scores on these scales indicate that patients and their families are in better condition in terms of daily living activities—functionality and biopsychosocial [12, 13]. Obtaining lower scores as the education level of the parents increases draws attention to the importance of education in combating the disease and its effects. It is expected that JAB‐Q will have significant results in the family evaluation. Finding significant results in the CHAQ—general evaluation is more remarkable in that it indicates the effects of the education level of the family on the child. Unfortunately, there is no study in the literature that describes any relationship between the CHAQ and parents' education level.

In a systematic review aiming to address the experiences of children with JIA and their families, it was concluded that the primary source of information about children's disease is parents [26]. However, parents may have difficulty in accessing the right information or transferring this information to their children. According to the same review, HL is a key factor for the development of effective self‐management. Self‐management skills are the main factors that ensure proper development and progress while living with JIA [26]. Acquiring sufficient knowledge and skills is important in terms of helping children and their parents cope with the disease, as well as gaining self‐confidence and the ability to live independently. As children grow older, they gradually take over the work of developing these self‐management skills from their parents. In this context, we believe that steps should be taken to improve HL for parents and children at an early stage. To make this planning, we emphasize the need for scales and studies that will evaluate HL.

About 50% of children with JIA preserve the disease actively into adulthood [26]. The concept of ‘transition readiness’ emerges during the transfer of paediatric patients to adult rheumatology outpatient clinics [27]. Although readiness for transition depends on many other parameters, we think that HL is important in this regard, as it is related to the decision‐making capacity of patients regarding their health status. We believe that if the HL is at a sufficient level, the transition to the adult polyclinic will be smoother and easier.

As the education level of the families increased, a positive change was observed in the 10‐SCT test results. No change was observed in the other two performance tests, suggesting that this is a clinically insignificant result.

Finally, the HL levels of the polyarticular group, which is one of the JIA subtypes, were found to be higher than the oligoarticular group. This may be because the oligoarticular group corresponds to about half of the patients.

The main limitation of the study is that it did not include a healthy group with the same demographic characteristics. While examining the HL levels of children belonging to a rare disease group is one of the superior aspects of the study, it may be more beneficial to consider the control group together. Another limitation is HL in paediatric patients, of which there are very few studies, so factors likely to affect this parameter could not be selected through the literature. This deficiency has been tried to be resolved by examining the demographic, clinical, daily life activities, biopsychosocial and physical activity status of children with JIA. In addition, conducting the study in a specific country may prevent international and general conclusions from being made. For these reasons, there is a need for more comprehensive and more studies on this subject.

## 5 Conclusions

According to the results of our study, children's HL levels show a positive increase as they get older, and there is no significant relationship between parents' HL levels. Parents' HL levels and education levels are compatible with the AHLS, which includes objective questions. As the education level of the families increases, positive changes are observed in the CHAQ and JAB‐Q parameters. In our study, it was found that the high education levels of the parents positively affected the quality of life and physical condition of their children and parental HL levels. In addition, it was shown that the HL levels of children with JIA were not statistically related to other parameters.

## Author Contributions

**Yahya Doğan:** conceptualization, data curation, formal analysis, investigation, methodology, project administration, writing–original draft, writing–review and editing. **Nur Banu Karaca:** conceptualization, data curation, formal analysis, investigation, methodology, project administration, writing–original draft, writing–review and editing. **Sinan Buran:** investigation, project administration, writing–review and editing. **Ege Nur Atabey Gerlegiz:** investigation, project administration, writing–review and editing. **Emil Aliyev:** investigation, project administration, writing–review and editing. **Yağmur Bayındır:** investigation, project administration, writing–review and editing. **Yelda Bilginer:** supervision, validation, writing–review and editing. **Edibe Ünal:** validation, supervision, writing–review and editing. **Seza Özen:** validation, writing–review and editing, supervision.

## Ethics Statement

The study was approved by the Ethical Committee of Hacettepe University (Approval: GO‐22/397). The authors confirm that their study's involvement with human subjects complies with the Declaration of Helsinki. Written informed consent was obtained from all participants before study enrolment.

## Conflicts of Interest

The authors declare no conflicts of interest.

## Data Availability

The data that support the findings of this study are available on request from the corresponding author. The data are not publicly available due to privacy or ethical restrictions.
